# A Study on the Modified Arrhenius Equation Using the Oxygen Permeation Block Model of Crosslink Structure

**DOI:** 10.3390/polym11010136

**Published:** 2019-01-14

**Authors:** Byungwoo Moon, Namgyu Jun, Soo Park, Chang-Sung Seok, Ui Seok Hong

**Affiliations:** Department of Mechanical Engineering, Sungkyunkwan University, Suwon-si, Gyeonggi-do 16419, Korea; moonmoon17@hanmir.com (B.M.); junng90@naver.com (N.J.); yabsabe@gmail.com (S.P.); sharperm@naver.com (U.S.H.)

**Keywords:** modified Arrhenius equation, degradation rate, crosslink density, oxygen permeation, nonlinear characteristics equation

## Abstract

Polymers are widely used in various industries because of their characteristics such as elasticity, abrasion resistance, fatigue resistance and low temperature. In particular, the tensile characteristic of rubber composites is important for the stability of industrial equipment because it determines the energy absorption rates and vibration damping. However, when a product is used for a long period of time, polymers become hardened owing to the changes in characteristics because of aging, thereby reducing the performance and increasing the possibility of accidents. Therefore, accurately predicting the mechanical properties of polymers is important for preventing industrial accidents while operating a machine. In general reactions, the linear Arrhenius equation is used to predict the aging characteristics; however, for rubber composites, it is more accurate to predict the aging characteristics using nonlinear equations rather than linear equations. However, the reason that the characteristic equation of the polymer appears nonlinear is not well known, and studies on the change in the characteristics of the natural and butadiene rubber owing to degradation are still lacking. In this study, a tensile test is performed with different aging temperatures and aging time to evaluate the aging characteristics of rubber composites using strain energy density. We propose a block effect of crosslink structure to express the nonlinear aging characteristics, assuming that a limited reaction can occur owing to the blocking of reactants in the rubber composites. Consequently, we found that a relationship exists between the crosslink structure and aging characteristics when the reduction in crosslink space owing to aging is represented stochastically. In addition, a modified Arrhenius equation, which is expressed as a function of time, is proposed to predict the degradation rate for all aging temperatures and aging times, and the formula is validated by comparing the degradation rate obtained experimentally with the degradation rate predicted by the modified Arrhenius equation.

## 1. Introduction

Rubber composites are widely used in industrial components because of their good energy-absorbing properties, resilience, elasticity and high extensibility. In particular, in a vibrating machine, the role of rubber that supports the body of the structure and absorbs vibrations and shocks generated from the ground is crucial. [1,2] In addition, the tensile characteristic of rubber composites determines the energy absorption rates and vibration damping, which is an important factor for the stability of an industrial equipment. However, when the product is used for a long period, rubber composites become hardened [3,4] owing to the changes in the characteristics because of degradation, thereby reducing the energy absorption rate and increasing the probability of accidents. Therefore, the evaluation of the mechanical properties of aged rubber composites has become important to prevent accidents while operating a machine [5]. Generally, the room-temperature aging test is adopted as a method of evaluating the degradation properties of rubber composites; however, this test takes a long time to evaluate the degradation properties. Hence, many researchers have conducted accelerated tests to evaluate the mechanical properties of rubber using the Arrhenius equation [6,7,8]. Kim et al. used the Arrhenius equation by substituting the compression set of ethylene propylene diene monomer (EPDM) rubber, which is used for motor fans for its characteristic value [9]. Han et al. analyzed the results obtained using the tensile strain of rubber hoses from manufacturing engine radiators as the characteristic value of the Arrhenius equation [10]. However, the parameters for the degradation characteristics of rubber composites appropriate for use in the Arrhenius equation are not well known; hence, research on these parameters should be performed in terms of reliability. In this study, we attempt to evaluate the degradation characteristics of rubber composites by performing tensile tests [11] on the rubbers at various aging temperatures and aging times. For this purpose, strain energy densities (SED) [12,13] were used and the tensile properties and SED values of rubber materials with varied degradation conditions were compared and analyzed [14]. 

In addition, we assumed that a limited reaction can occur owing to the blocking of the reactants in the rubber composite, and proposed an oxygen permeation block model of the crosslinked structure to express nonlinear aging characteristics. Consequently, a relationship between the crosslink structure and aging characteristics was found when the reduction in crosslink space owing to aging was represented stochastically. Thus, we suggest a modified characteristic equation and nonlinear Arrhenius equation, which is expressed as a function of time. By applying the modified Arrhenius equation, we derived the relationship between short-term high-temperature aging and long-term low-temperature aging, and suggest a method for predicting the degradation rate of rubber composites under all aging conditions. Finally, the accuracy of the formula was demonstrated by comparing the actual experimental results obtained using an aged specimen with the calculated degradation rates predicted by the modified Arrhenius equation.

## 2. Evaluation of Tensile Properties of Aged Specimen

### 2.1. Tensile Tests

The primary materials of the polymer used in this study were a containing 50% natural rubber (NR) and 50% butadiene rubber (BR). The shape of the specimens used for the tensile test was a dumbbell type No. 3, according to KS M 6518. The specimen was made by die cutting a 2 mm rubber sheet. A blade meeting the KS standard was used for cutting. The cut specimens were degraded at high temperatures using an environmental chamber (within a 1 °C temperature error). The conditions for the degradation procedure are listed in Table 1.

An electro hydraulic universal testing machine was used for the tensile test, and a 200 N load cell, suitable for a load of a rubber tensile test, was installed and tested. The overall testing process was performed according to the ASTM D412-a [11] tensile test specifications for vulcanized rubbers and thermoplastic elastomers. Before starting this test, the strain range to be tested was repeated 30 times to stabilize the rubber molecular structure, and Mullin’s effect [15] was removed.

### 2.2. Results of the Tensile Test

**Figure 1:**
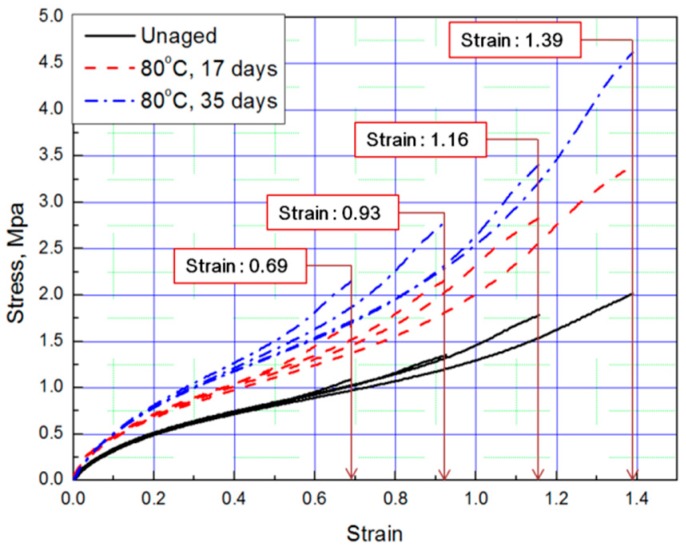
Tensile curves according to aging time (Strain: 0.69, 0.93, 1.16, 1.39).

**Figure 2:**
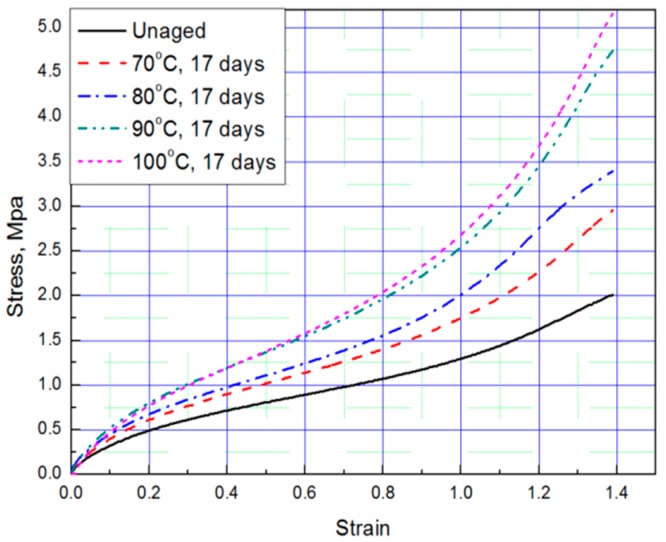
Tensile curves according to aging temperature (Strain: 1.39).

Tensile tests were conducted to obtain stress–strain curves with four strain values (strain: 0.69, 0.93, 1.16, 1.39) of aged rubber composites, and the results are plotted in Figure 1 and Figure 2. The results of the tensile test, based on the aging time and aging temperature, indicate that as the degradation of the rubber progressed, it hardened and the stress increased under the same strain condition. Thus, we conclude that the unaged rubber composites specimens are the softest, and the degradation specimens become stiffer as aging time and temperature increased.

### 2.3. Derivation of SED-strain relationship

**Figure 3:**
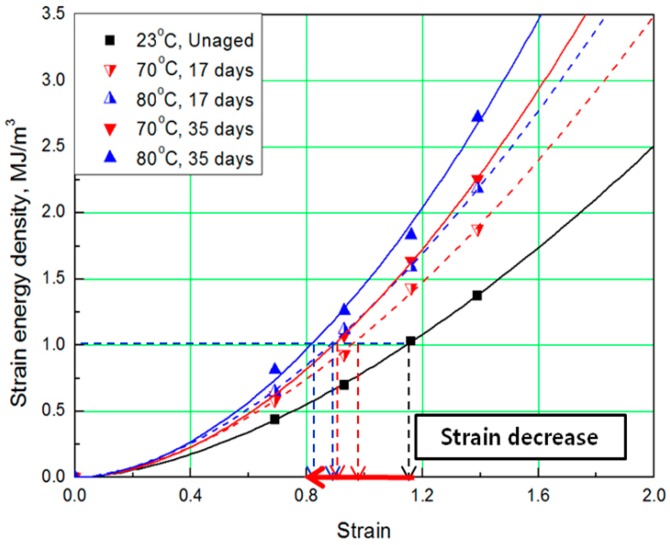
Strain energy density (SED)-strain curves according to aging time.

**Figure 4:**
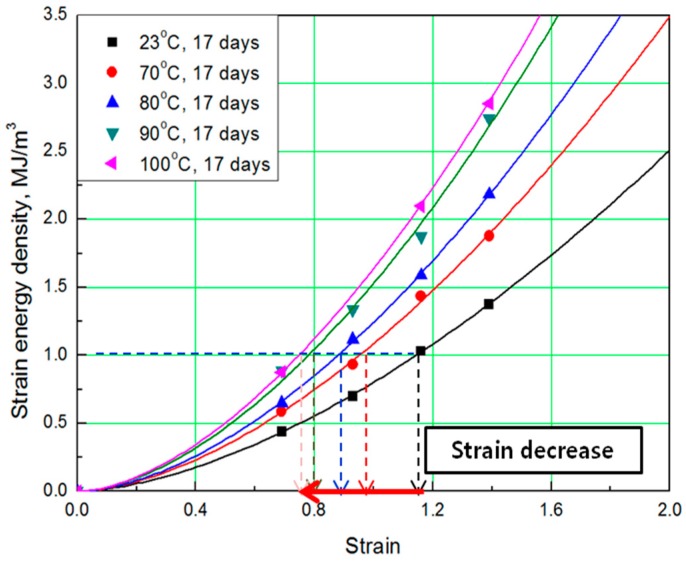
SED-strain curves according to aging temperature.

To determine the strain energy density, the lower area of the stress-strain curve must be calculated. Therefore, Simpson’s rule [16], which is suitable for calculating the area of a stress-strain curve exhibiting nonlinear behavior, was applied to the equation. The SED-strain equation (as shown by Equation (1)) for the aged specimens was determined by fitting the calculated SED value. In this case, ε represents strain of the rubber, *a* represents coefficient number, and *b* is an exponential term [14]. The coefficients, according to aging conditions, are listed in Table 2; the strain values at the same SED (1 MJ/m^3^) were obtained as shown in Figure 3 and Figure 4.

(1) $$SED=a\times {\left(\varepsilon \right)}^{b}$$

**Figure 5:**
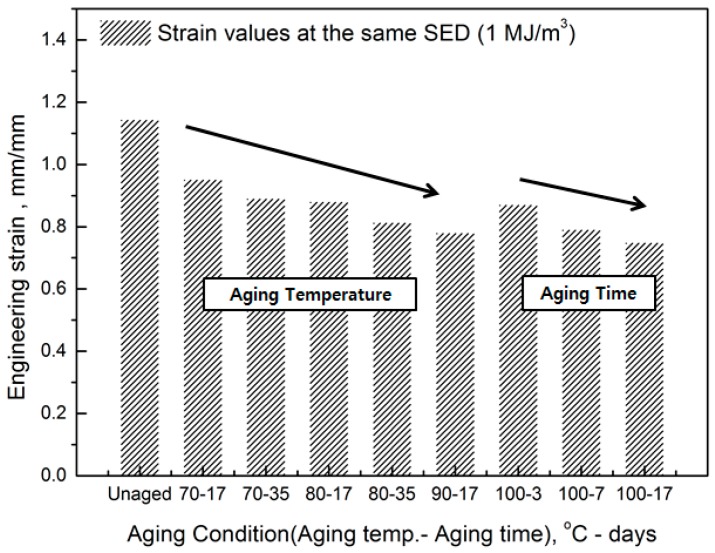
Decrease in strain on aging.

The comprehensive results of the evaluation of rubber composites are as follows. As degradation progresses, the rubber composites become hardened and the slope of the tensile strength increases. In addition, the strain tends to decrease at the same SED (1 MJ/m^3^), depending on the increase in aging temperature and time (Figure 5). Therefore, we regard the strain value for this SED (1 MJ/m^3^) as the characteristic value and the degradation rate of rubber composites as the strain reduction rate.

## 3. Application of Modified Arrhenius Equation

### 3.1. General Arrhenius Equation

The characteristic equation of the reaction rate constant *k* used for the Arrhenius equation is generally expressed as Equation (2).

(2) $$-\frac{dP}{dt}=kP,\;\ln\left[\frac{P}{P_{0}}\right]=-kt$$

In this case, *P* represents the characteristic value of the rubber, *P_0_* represents an initial characteristic value, *t* represents time, and *k* is a reaction rate constant. If the lifetime of the rubber is defined as the time until the characteristic value becomes *P* from Equation (2), the lifetime (*t*) can be expressed by Equation (3) at that time.

(3) $$t=-\;\ln\left[\frac{P}{P_{0}}\right]/k$$

The reaction rate constant *k* from Equation (2), a value indicating a degradation reaction, can be expressed by Equations (4) and (5) using the Arrhenius equation. At time *t*, *A* and *C* are constants, *E_a_* is the activation energy (J/mol), *R* is a gas constant (8.314 J/mol∙K), and *T* is the absolute temperature.

(4) $$k=A\cdot e^{-E_{a}/RT}$$

(5) $$\ln k=-\frac{E_{a}}{RT}+C$$

The lifetime *t* in Equation (3) can be calculated using the empirical Arrhenius equation (Equation [4]) because the equations represent the relationship between lifetime and temperature. Thus, the lifetime can be converted into temperature. With the characteristic value of *P*, lifetime *t_1_* is derived at temperature *T*_1_, and lifetime *t_2_* is derived at temperature *T*_2_. Consequently, Equation (6) can be obtained.

(6) $$\ln\left[\frac{t_{1}}{t_{2}}\right]=\frac{E_{a}}{R}\left[\frac{1}{T_{1}}-\frac{1}{T_{2}}\right]$$

**Figure 6:**
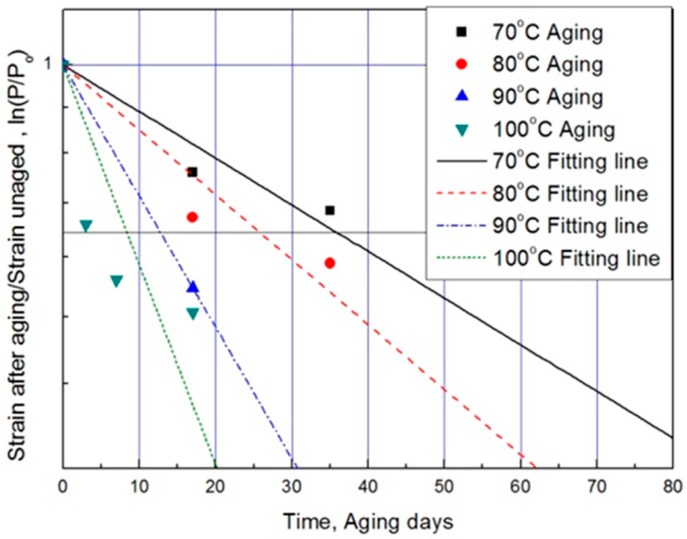
Characteristic equation according to aging time by temperature.

**Figure 7:**
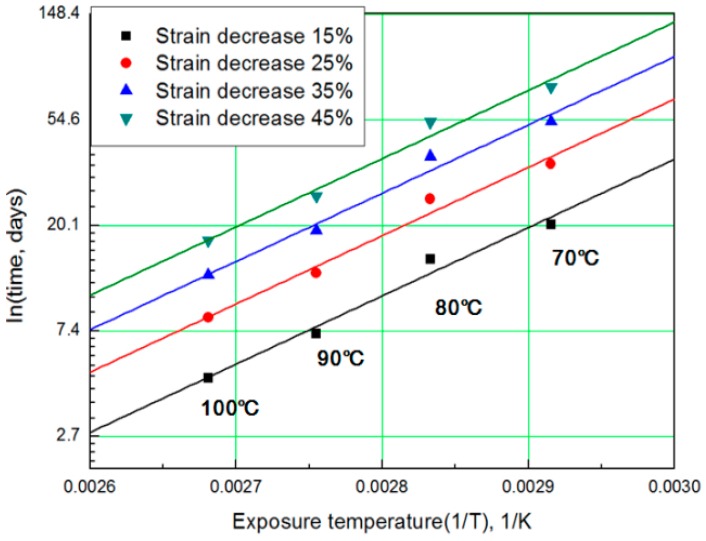
General Arrhenius plot according to the characteristic value.

For each aging temperature (70, 80, 90,= and 100 °C), the relationship between aging time and characteristic values is shown in Figure 6, and the reaction rate constant (*k*) of the characteristic equation is derived and listed in Table 3. When the temperature is low, the reaction rate constant *k* is small and the characteristic value changes gradually. However, as the temperature increases, the reaction rate increases and the characteristic value changes sharply. In this study, the aging time (*t*) is calculated at 15, 25, 35, and 45% reductions from the characteristic values using the characteristic equation. The results are plotted in Figure 7. The condition at a 25% decrease in characteristic value is derived as a function of temperature and time in Equation (7), and compared with the actual value.

(7) ln(t)=6472.6/T−15.2

The results of calculating the equivalent aging time, converted from the aging temperature using Equation (7), are shown in Table 4. The mean deviation between the predicted characteristic value using the characteristic equation and the actual experimental value (Figure 6) is 42% or more. Therefore, if we convert the characteristic values into aging time by applying the general Arrhenius equation, a large difference occurs in the resulting values [17]. For example, when the degradation condition caused by decreasing the characteristic value by 25% is calculated with the Arrhenius equation, it becomes nine days at 100 °C (Table 4). However, as the actual specimen is equivalent to a specimen aged at 100 °C for approximately three days, the difference is almost a factor of three. Depending on the difference in these results, ISO 11346 [18] suggests using fitting functions of the logarithmic scale or establishing characteristic equations as suitable expressions. However, hitherto, standard characteristic equations for rubber are few. Thus, most studies pertaining to the degradation life of rubber composites used general characteristic equations with large deviations. Other researchers have substituted certain characteristic values regardless of the degradation effect and acquired an Arrhenius equation by analyzing the relationship between aging temperature and aging time [19,20]. In this study, we attempt to determine mathematical expressions that can predict the degradation rate of rubber composites under all aging conditions, using the modified characteristic equation and the Arrhenius equation.

### 3.2. Oxygen Permeation Block Model

As a result of predicting the aging properties by applying the linear Arrhenius equation, the difference between the experimental and theoretical values was significant. Therefore, when developing products using linear Arrhenius equations, it is difficult to predict changes owing to aging, and product characteristics can often vary when used for long periods of time. To solve this nonlinear [21] aging behavior problem, researchers have analyzed the aging behavior of the polymer by dividing the aging period of the polymer into linear sections of 2–3 stages. However, because polymer aging requires a wide temperature range and high test sensitivity, empirical extrapolation could not solve the essential problem. In 2000, Dakin’s kinetic equation shows the reaction of the polymer and the calibrated activation energy that varies with the deterioration of the material [22]. Using this, an individual *E_a_* was set in the reaction at a high temperature and a low temperature. However, it is only a numerical conversion approach using the test results. In 2005, nonlinear Arrhenius [23,24] behavior was studied to accurately predict the aging characteristics of polymers. Celina et al. presented a nonlinear Arrhenius equation with two reactions, assuming that the reaction rate exhibits temperature dependence [25]. This method does not require complex kinetic modeling, easily determines the individual activation energies and demonstrates excellent compatibility by representing at least two reactions. However, the primary reason that activation energy appears nonlinearly is not suggested. Many studies have emphasized the importance of changes in mechanical properties and activation energies, but they have not yet found the fundamental cause of the nonlinear reaction rate constant of characteristic values.

**Figure 8:**
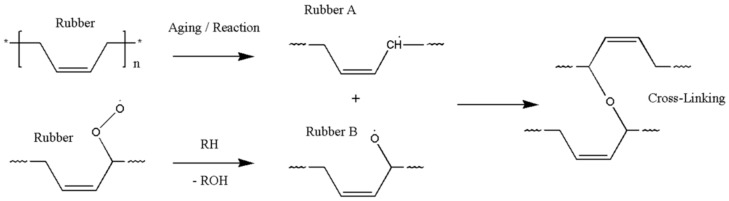
Crosslinking mechanism.

**Figure 9:**
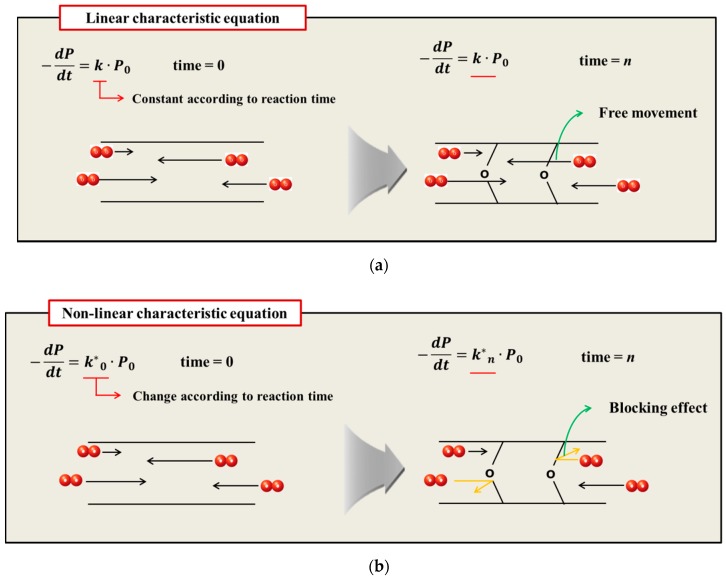
(**a**) General aging reaction; (**b**) Aging reaction according to the oxygen permeation block effect of crosslink structure.

The oxidative hardening reaction of the polymer owing to aging, and the increase in the crosslinking density are shown in Figure 8. At this time, Bernstein et al. showed that the aging rate is related to the consumption of oxygen by analyzing the relation between the oxygen consumption measurement and the reaction rate of the polymer [26,27,28]. Thus, the reason that the reaction rate of the polymer decreases as the aging progresses is because the probability of the rubber reacting with oxygen is reduced. In this study, the reason that the reaction rate decreases as the aging progresses, and the difference between the linear Arrhenius equation and the nonlinear Arrhenius equation are suggested as follows. In the case of gas and liquid, oxygen molecules diffuse freely between reactants, as shown in Figure 9a. Therefore, the reaction rate constant is represented by a linear equation that is independent of time (*t*) by a continuous reaction, and a general Arrhenius equation is established. However, in the case of rubber molecules, the crosslinking structure increased by aging interferes with the permeation of oxygen [29,30], as shown in Figure 9b, and the reaction of the molecules is inhibited over time. Therefore, we suggest a modified characteristic equation and nonlinear Arrhenius equation, which is expressed as a function of time.

### 3.3. Modified Arrhenius Equation

In a general characteristic equation, the properties decrease linearly in proportion to the reaction rate constant and time in accordance with Equation (2). However, in the case of the actual test results on the rubber composites shown in Figure 6, as aging time increases, the reduction rate of the characteristic value decreases. Thus, if an existing characteristic equation is used, a large error occurs. Because of the above reasons, other researchers have used the non-Arrhenius equation by substituting the accelerative shift factors for the individual activation energy [25,31]. However, it is only a numerical conversion approach using the test results. Further, the fundamental parameters of the degradation characteristics for rubber composites in the Arrhenius equation are not well known [32]. In this study, we formulated the relationship that the reaction rate constant is inversely proportional to the time based on the oxygen permeation block model. Therefore, a modified characteristic equation is expressed as Equation (8), and the modified Arrhenius equation is derived as follows by substituting the time term for the characteristic equation.

(8) $$-\frac{dP}{dt}=k^{*}{}_{n}P_{0}$$

where $k^{*}{}_{n}=k^{*}/\left(t+1\right)$.

By integrating Equation (8), we can obtain Equation (9) as follows:

(9) $$-\int_{P_{o}}^{P}\left(\frac{1}{P_{0}}\right)dP=k^{*}\int_{t_{0}}^{t}\frac{1}{\left(t+1\right)}dt\;\frac{P}{P_{0}}=1-k^{*}\times \left[\ln\left(t+1\right)-\ln\left(t_{0}+1\right)\right]$$

If the initial value at aging time 0 is substituted for *t_0_*, the modified characteristic equation is derived as Equation (10).

(10) $$\frac{P}{P_{0}}=1-k^{*}\times \ln\left(t+1\right)$$

In addition, the modified Arrhenius equation in which the activation energy and the constant are expressed as a function of the characteristic value is presented as Equation (11).

(11) $$k^{*}=A^{*}\cdot e^{-E_{a}^{*}/RT}$$

when Equation (10) is substituted into the modified Arrhenius equation (Equation (11)), k* is eliminated and the result is expressed as Equation (12).

(12) $$\ln\left[1-\frac{P}{P_{0}}\right]=-\frac{E_{a}^{*}}{RT}+C+\ln\left(t\right)$$

where $A^{*}=f\left(t,\frac{P}{P_{0}}\right)$, $C=f_{1}\left(\frac{p}{p_{0}}\right)$, $E_{a}^{*}=f_{2}\left(\frac{p}{p_{0}}\right)$.

Finally, in the case of the characteristic value *P*, time *t_1_* at temperature *T*_1_ can be represented as equal to time *t_2_* at temperature *T*_2_, which is expressed as Equation (13).

(13) $$\ln\left[\frac{t_{1}}{t_{2}}\right]=\frac{E_{a}^{*}}{R}\left[\frac{1}{T_{1}}-\frac{1}{T_{2}}\right]$$

**Figure 10:**
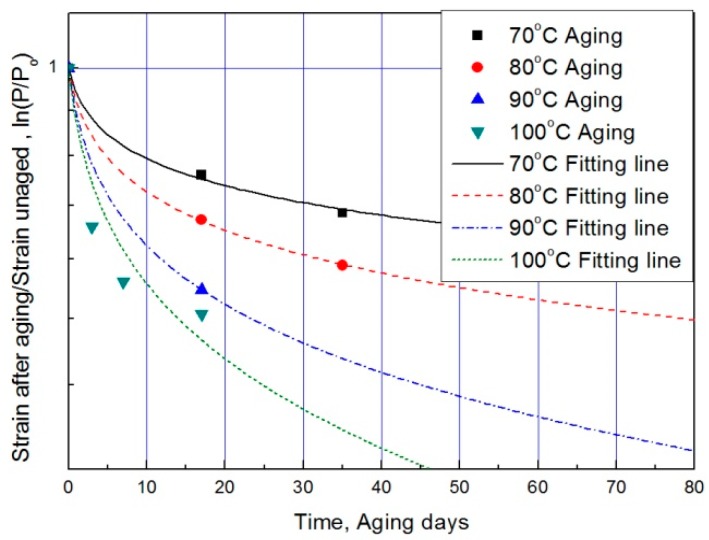
Modified characteristic equation according to aging time by temperature.

**Figure 11:**
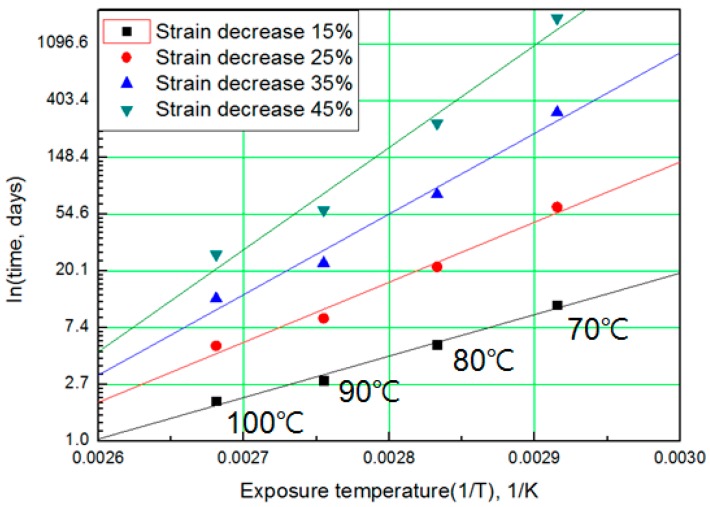
Arrhenius plot according to characteristic values.

**Figure 12:**
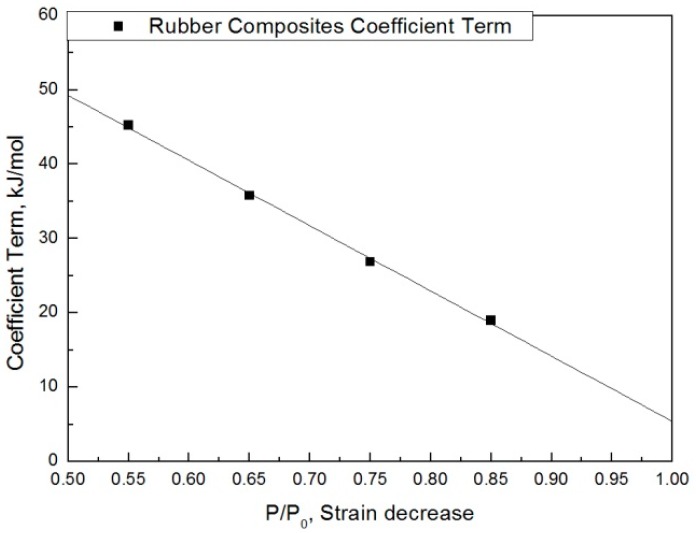
Coefficient term according to characteristic value.

**Figure 13:**
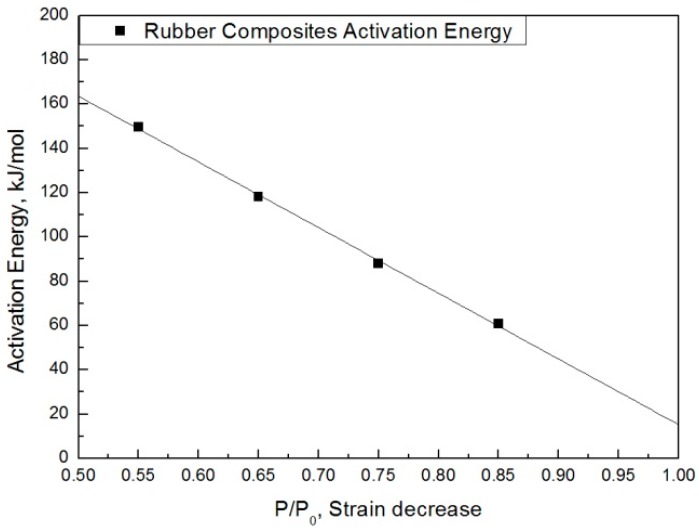
Activation energy according to the characteristic value.

The modified characteristic equation (Equation (10)) is applied to derive the reaction rate constant, and the results are presented in Table 5. As a result of the derivation, the k* value of the reaction rate increases with increasing temperature, and the characteristic value decreases significantly at the same aging time. As the aging time increases, the reduction rate of the characteristic value decreases. These results are shown in Figure 10. An analysis of the data shows that the mean deviation of the values predicted by the experiment and the modified characteristic equation decrease significantly to within 17%. In addition, the difference between the two results is less than 4% when the upper usage temperature limits (100 °C) for the NR compound is excluded. The temperatures and times required for the characteristic value to decrease by 15, 25, 35, and 45% are shown in Figure 11. By applying the modified Arrhenius equation (Equation (11)), the activation energy (E_a_*) and constant (*C*) were obtained. Further, the results based on the characteristic values, are represented in Figure 12 and Figure 13. The activation energy (E_a_*) and the constant (*C*) both indicated a linear relation to the characteristic value, and regression analysis was used to derive their respective Equations, *i.e*., (14) and (15). Finally, the modified Arrhenius equation was derived as Equation (16). The Arrhenius expression with the characteristic value is expressed as Equation (17).

(14) $$C=f_{1}\left(\frac{p}{p_{0}}\right)=-87.73\times \frac{p}{p_{0}}+93.13$$

(15) $$E_{a}^{*}=f_{2}\left(\frac{p}{p_{0}}\right)=-296.7\times \frac{p}{p_{0}}+311.86$$

(16) $$\ln\left(t\right)=f_{2}\left(\frac{p}{p_{0}}\right)/RT-f_{1}\left(\frac{p}{p_{0}}\right)$$

(17) $$F\left(T,t\right)=\frac{P}{P_{0}}=\frac{RT\cdot \left(\ln\left[t\right]+93.13\right)-311.86}{87.73\cdot RT-296.7}$$

### 3.4. Verification and Application of Modified Arrhenius Equation

**Figure 14:**
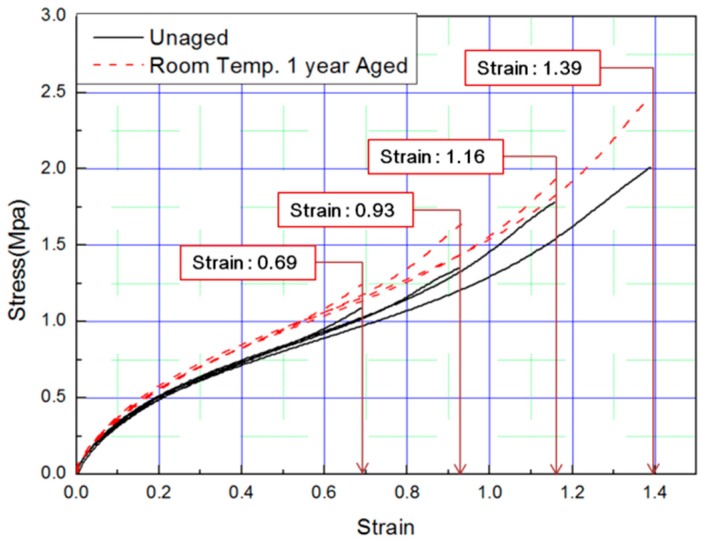
Tensile curves for specimen aged for one year.

**Figure 15:**
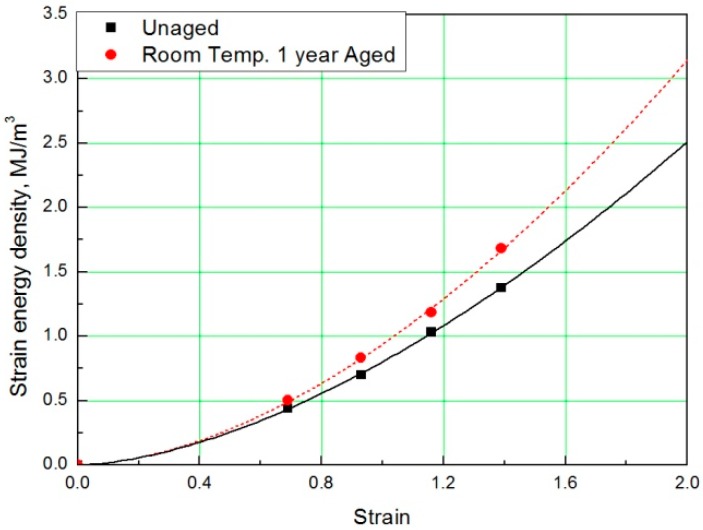
SED-strain curves for specimen aged for one year.

In this study, to verify the modified Arrhenius equation, an additional tensile test was conducted on specimens aged at room temperature for one year. Room temperature aging was conducted in a laboratory with a temperature distribution of 8 °C to 25 °C, and the test specimen was stored in the shade without exposure to sunlight. Even in the room temperature aging test, the rubber composites become hardened and the stress increased in a manner equivalent to the accelerated aging test results. It indicated the same tendency that the strain value decreases at the same SED, as shown in Figure 14 and Figure 15. Finally, the errors were analyzed by comparing the characteristics of the rubber composites obtained experimentally and the calculated values of the modified Arrhenius equation; the results are shown in Table 6. The average error between the experimental values and the predicted values for nine degradation conditions indicate a high accuracy of 3%; however, in the case of the room temperature aging test, an error of 5.3% occurred. This is because we used 17 °C as room temperature, and this value was the average value of the temperature distribution (8 °C to 25 °C), to calculate the characteristic value obtained from the modified Arrhenius equation. It is expected that if the temperature of the laboratory during the room temperature aging test is maintained constant and an accurate temperature is used in the modified Arrhenius equation, the error will decrease. In this study, the method to obtain the degradation rate of rubber composites under all conditions was presented by Equation (17). Additionally, an equivalent degradation conversion formula, which can convert short-term high-temperature aging into long-term low-temperature aging, was derived using Equation (13). The accelerated conditions at room temperature for one year are presented in Table 7.

## 4. Conclusions

The aim of this study is to determine the fundamental parameters for the degradation properties of rubber compounds, suitable for the Arrhenius equation. In this study, an oxygen permeation blocking model was proposed to predict the degradation rate of rubber composites. In this process, a tensile test was performed with the different aging conditions to evaluate the degradation characteristics using strain. Consequently, it was confirmed that as degradation progressed, the rubber composites become hardened, and the strain decreased at the same SED (1 MJ/m^3^). Here, we assumed that the crosslinked structure interfered with the reaction of oxygen and rubber as the aging progressed. Thus, the modified Arrhenius equation was derived by adding the degradation time term to the reaction rate constant of the characteristic equation. Finally, the validity of the formula was verified by comparing the degradation rate obtained by experimentally with the degradation rate predicted by the modified Arrhenius equation, and the following conclusions were obtained:

1 In a general characteristic equation, the properties decreased linearly in proportion to reaction rate constant and time. However, in most cases, the reaction rate of the characteristic value decreased as the aging time increased. The reason that the reaction rate of the polymer decreased as the aging progressed was because the probability of the rubber reacting with oxygen was reduced. In the case of rubber molecules, the crosslinking structure increased by aging interfered with the permeation of oxygen, and the reaction of the molecules was inhibited over time. Therefore, we formulated a relationship where the reaction rate constant was inversely proportional to time based on the experimental results. The modified characteristic equation was proposed as a function of time, and the modified Arrhenius equation was derived by substituting the time function for the characteristic equation.
2 In the case of the general Arrhenius equation, the resulting average deviation between the calculated and experimental values was 42% or more. However, in the case of a modified characteristic equation as a function of time, we observed that the average deviation in the experimental and calculated value decreased considerably to within 17%. Consequently, comparisons of the nine experimental values obtained with different degradation conditions with the predicted values indicated that the accuracy of the modified Arrhenius equation was relatively high, with an average error of 3%. Thus, using a modified Arrhenius equation derived from an oxygen permeation block model could predict the aging behavior of rubber materials accurately.
3 By applying the modified Arrhenius equation, we derived the relationship between short-term high-temperature aging and long-term low-temperature aging, and suggested a method for predicting the degradation rate of rubber composites under all aging conditions. Therefore, it was possible to accurately predict changes in the characteristics of the rubber composites by performing the acceleration test, and the energy absorption rate and stability that changed with degradation rate could be considered quickly in the design stage.

## Figures and Tables

**Table 1:** Accelerated degradation conditions for rubber composites.

Degradation Condition					
Aging temp. (°C)	23	70	80	90	100
Aging time (Day)	-	17, 35	17, 35	17	3, 7, 17

**Table 2:** Coefficients of SED strain curves.

Aging (°C, day)	Coefficient Number, *a*	Exponential Term, *b*
Unaged	0.802	1.647
70, 17	1.088	1.685
70, 35	1.238	1.835
80, 17	1.244	1.706
80, 35	1.462	1.836
90, 17	1.530	1.709
100, 3	1.289	1.816
100, 7	1.515	1.764
100, 17	1.639	1.700

**Table 3:** Reaction rate constant (*k*) depending on aging temperature.

Aging (°C)	70	80	90	100
Reaction rate constant (*k*)	0.00801	0.01115	0.02253	0.03430

**Table 4:** Aging days conversion results.

Property (*P*/*P_0_*)	Equivalent Degradation Conditions	
	T (°C)	Days
0.75	23	786
	70	39
	80	23
	90	13
	100	9

**Table 5:** Reaction rate constant (k*) depending on aging temperature.

Aging (°C)	70	80	90	100
Reaction rate constant (k*)	0.060	0.080	0.110	0.135

**Table 6:** Results of degradation conversion.

Aging Condition		Normalized Property Value		Error
*T* (°C)	Days	Experimental	Predicted	
70	17	0.832	0.824	1.0%
70	35	0.779	0.779	0%
80	17	0.770	0.773	0.4%
80	35	0.711	0.719	1.1%
90	17	0.682	0.699	2.5%
100	3	0.761	0.802	5.4%
100	7	0.691	0.695	0.6%
100	17	0.654	0.582	11.0%
17 (Room)	365	0.907	0.859	5.3%

**Table 7:** Equivalent degradation conversion results.

The Equivalent Degradation Conversion Table					
***T* (°C)**	17	40	60	80	100
**Day**	365	65	17.5	5.4	1.9
